# Evaluation of the *in vitro* antioxidant and antitumor activity of hydroalcoholic extract from *Jatropha mollissima* leaves in Wistar rats

**DOI:** 10.3389/fchem.2023.1283618

**Published:** 2023-12-18

**Authors:** Muhammad Omer Iqbal, Yuchao Gu, Imran Ahmad Khan, Ruihong Wang, Jin Chen

**Affiliations:** ^1^ College of Marine Science and Biological Engineering, Qingdao University of Science and Technology, Qingdao, China; ^2^ Key Laboratory of Marine Drugs, The Ministry of Education, School of Medicine and Pharmacy, Ocean University of China, Qingdao, China; ^3^ Fatima Tu Zahara Department of Life Sciences, Muhammad Institute of Medical and Allied Sciences, Multan, Pakistan; ^4^ Department of Pharmacy, MNS University of Agriculture, Multan, Pakistan; ^5^ The Affiliated Qingdao Central Hospital of Qingdao University, Qingdao, China

**Keywords:** antioxidant, cardioprotective, nephroprotective, doxorubicin, anticancer, *Jatropha mollissima*

## Abstract

**Introduction:** Despite modern sciences and advancements in new drugs or chemicals, the new era now rushes natural remedies for various illnesses and diseases that lead to end organ damage. In this study, we investigated *Jatropha mollissima* ethanolic extract’s effect against doxorubicin-induced cardiotoxicity and renal toxicity.

**Methods:** To determine phytochemicals, a phytochemical screening was conducted. Various assays were used to measure the antioxidant activity, including the DPPH (2,2-diphenylpicrylhydrazyl), SOD (superoxide dismutase), NO (nitric oxide), and others. The antiproliferative effect of *Jm* was assessed by MTT assay; morphological analysis was performed using an inverted and phase contrast microscope, ultra morphological analysis of apoptosis with acridine orange (AO)/propidium iodide (PI) staining.

**Results:** It was seen that doxorubicin caused elevated serum markers and abnormal changes in histological patterns. The significant reduction in cardiac and renal marker levels seen in groups given either 400 or 600 mg/kg of crude extract demonstrates that *Jm* has a protective effect against doxorubicin-induced cardiotoxicity due to the presence of active phytoconstituents having antioxidant potential. There is a dose-dependent decrease in cell viability when using *J. mollissima*. Apoptosis was observed in the treated cells.

**Conclusion:** In conclusion, our research lends credence to the idea that *J. mollissima* could be used for cancer management and have cardioprotective and nephroprotective effects.

## 1 Introduction

Doxorubicin, a type of anthracycline, was first isolated in the 1960s from the pigment-producing bacterium *Streptomyces* peucetius. Doxorubicin works by inhibiting topoisomerase II, which increases the amount of cleavable enzyme-DNA linked complexes during DNA replication and thus blocks the ligation of nucleotide strands after double-strand breaks. Moreover, it inhibits protein synthesis and prevents DNA replication by intercalating between base pairs in the DNA helix. Anthracycline is the most effective anti-cancer drug used for the treatment of breast, lung, gastric, ovarian cancer, pediatric cancers, hematologic malignancies, soft tissue sarcomas, Wilms’ tumor, neuroblastoma, bone sarcomas, transitional cell bladder carcinoma, bronchogenic carcinoma, and thyroid carcinoma (Kirstein, 2009). The major toxicity caused by doxorubicin includes cardiomyopathy, renal toxicity, hepatotoxicity (Ghigo et al., 2016).

Multiple mechanisms of doxorubicin produce cardiotoxicity; however, the exact mechanism is still controversial, which includes the formation of ROS occurs due to oxidative stress by Cytochrome P450 reductase, which causes a reduction of the quinone form of doxorubicin into free radical semiquinone form in mitochondrial cells. These semi-quinine forms are rapidly oxidized into quinine by producing superoxide anions, which convert into oxygen and generate hydrogen peroxide, which turns into superoxide free radicals by ferrous ions. This superoxide-free radical build up in myocytes, causing lipid peroxidation, which destroys the mitochondrial membrane, endoplasmic reticulum, and nucleic acid. Mitochondrial cells are abundantly present in cardiac cells and more susceptible to free radical toxicity (Salazar-Mendiguchía et al., 2014), inhibition of synthesis of macromolecules is caused by DNA intercalation (Outomuro et al., 2007), DNA alkylation, DNA cross-linking, Protein and nucleic acid synthesis are stifled, expression of several vasoactive amines, including prostaglandins, histamine, etc., Transmission of adenosine triphosphate (ATP) production within cardiac cells results from adenylate cyclase activity alterations. Doxorubicin also causes elevated levels of nitric oxide (NO) by stimulation of endothelial NOS (eNOS) expression and superoxide formation by enhancing the activity of endothelial proteins. The eNOS has a key role in cardiotoxicity by initiating ROS apoptosis of endothelium, calcium overload in ventricular myocardium, induction of apoptosis causing DNA fragmentation and cell shrinkage by inhibiting RNA polymerases and oxidative damage, lysosomal changes, alteration in Na+/K+ adenosine triphosphate (ATPase) pump and Ca^2+^ ATPase pump (Ruggeri et al., 2018) and Myocytes suffer iron-dependent oxidative damage due to the formation of semiquinone metabolites that cause the delocalization of Fe (II) from ferritin, leading to the production of hydrogen peroxide, which in turn sustains the generation of hydroxyl radicals and lipid peroxidation (Ma et al., 2013).

Doxorubicin-producing free radicals have a more damaging effect on the heart due to fewer antioxidants like superoxide dismutase, glutathione, and catalase than other organs like the kidney and liver. However, it causes damaging effects on kidney and liver but to a lesser extent than the heart because the heart contains cardiolipin. Mitochondrial membrane phospholipids with a high affinity for DOX accumulate in cardiac cells (Yancy et al., 2013).

Doxorubicin damages the kidney, causing lipid peroxidation and generation of ROS (reactive oxygen species), which causes activation of NF-KB, thus stimulating nitric oxide and TNF-α which causes nephrotoxicity, increase in hydroxyproline in the kidney and increases protein level in the urine (Chen et al., 2016). Lipid peroxidation and kidney damage are caused by the byproducts of NADPH-Cytochrome P-450’s conversion of doxorubicin to the semiquinone free radical, which in turn generates hydroxyl radical and superoxide anion (Nagai et al., 2018). Renal damage was also reported due to doxorubicin, which causes iron-dependent oxidative damage to renal cells by forming semiquinone metabolites that delocalize Fe (II) from ferritin that produce hydrogen peroxide and form hydroxyl radicals and lipid peroxidation and elevated levels of serum albumin, serum creatinine, and BUN. Previous studies also reported high MDA levels (Ahmed et al., 2019).

Given the rich diversity of plant life in Pakistan, this study set out to identify medicinal plants with cardioprotective and nephroprotective properties. Herbal medications are among the natural chemicals being studied by scientists because of their less negative effects. These plants can potentially provide low-cost, high-yield therapeutic options for the conditions mentioned above (Hosseini and Sahebkar, 2017). Cancer is a leading cause of death worldwide. Traditional therapy based on plant extracts has received renewed interest in the fight against cancer after being neglected for centuries. The wide variety of phytochemical substances found in plants and their relatively few side effects have led to a renewed interest in their application in cancer prevention and treatment in recent years (Kelloff, 1999).

In traditional medicine, the plant species *Jatropha mollissima* (Euphorbiaceae) is used for a wide variety of purposes, including as an anti-inflammatory remedy (de Queiroz Neto et al., 2019), antibacterial treatment (Bastos-Cavalcante et al., 2020), antioxidant protection (Dias et al., 2019), anthelmintic action (Abogmaza et al., 2020), kidney protection (Iqbal and Yahya, 2021; Omer Iqbal et al., 2021), liver protection (Iqbal et al., 2022), heart protection (Iqbal et al., 2021), and appetite suppression (de Albuquerque et al., 2007). The significance of *J. mollissima* in counteracting anxiety and depression has not been studied, despite the many research documenting the plant’s beneficial effects on health. The purpose of this research is to determine if *J. mollissima* has *in vitro* antioxidant and antitumor activity.

## 2 Methodology

### 2.1 Plant collection

*Jatropha mollissima* was purchased fresh from Multan, Pakistan. Bahauddin Zakariya University’s Botany Department Taxonomists validated the species and assigned a voucher number (R.R. Stewart F.W. Pak.725/16) for future reference.

### 2.2 Preparation of crude extract

Separated *J. mollissima* plants were sun-dried for 15 days before ground into a powder in a blender. It was first ground using an electric grinder before being submerged in a mixture of 70% ethanol and 30% distilled water for nine period of 3 days while being periodically shaken and agitated. Muslin fabric was used for preliminary coarse filtering before Whatman filter paper (NO.1) was used for final polishing.

The filtrate was then evaporated in a rotary evaporator (Heidolph Laborota 4000 efficient, Germany) at 30°C–40°C under vacuum. The filtrate was dried in an incubator until it reached a semi-solid consistency, then placed in a refrigerator (−40°C) for later use.

### 2.3 Chemicals and reagents and cell line

Doxorubicin, formalin, ketamine (Indus Pharma Lahore), xylazine (Prix Pharmaceutical Lahore), and ether were all utilized as analytical grade chemicals in scientific studies. DPPH, ferric chloride, 2-deoxyribose, NADH, nitroblue tetrazolium (NBT), and phenazine methosulfate (PMS) 3-(4,5-dimethythiazol-2-yl)-2,5-diphenyl tetrazolium bromide (MTT), were purchased from Sigma-Aldrich; Merck KGaA, respectively. The hepatocellular carcinoma (HepG2) cell line was used in this study.

### 2.4 Experimental animals and conditions

Male Wistar Albino rats weighing between 250 and 320 g were purchased from the animal house at the Muhammad Institute of Medical Allied Sciences in Multan. In the Pharmacology Research Laboratory at the Muhammad Institute of Medical and Allied Health Sciences, the animals were kept in polycarbonate cages with raw dust that was changed every 3 days in a standard laboratory setting (27°C ± 2°C). The rats had unlimited access to water and standard diet pallets. According to the National Research Council (Touitou et al., 2004), the research projects were approved by the Muhammad Institute of Medical and Allied Science’s Ethical Committee in Multan, Pakistan. A valid MIMAS/07-21/25261 authorization number was provided.

### 2.5 Study design

Twenty-four rats of the Wistar strain were split into four groups of six.

The first group (the control group) received saline solution without any additives.

Doxorubicin was administered to a group designated as “Group 2.”

Group 3 was given Doxo + *Jm* extract (400 mg/kg).
Group 4 was given Doxo + given (600 mg/kg) of *Jm* extract.

The first group (the control group) received saline solution 4 mL/kg for 21 days via nasal gastric tubing. Group 2 was the Doxorubicin group intoxicated with doxorubicin 10 mg/kg given intraperitoneal single bolus dose and N/S 4 mL/kg for 21 days via gastric tubing. Groups 3 and 4 received *Jm* extract treatments of 400 and 600 mg/kg, respectively, orally for 21 days, and on first day these groups received intraperitoneal injections of doxorubicin.

### 2.6 Preliminary phytochemical evaluation

Aqueous-ethanolic (30:70) extract of *J. mollissima* was evaluated for the possible presence of vital phytochemical classes using standard protocol (Ain, 2022).

### 2.7 Antioxidant activity

Antioxidant activity was performed using 2,2-diphenylpicrylhydrazyl (DPPH) and Nitric oxide (NO) assay, SOD and peroxide.

#### 2.7.1 2,2-diphenylpicrylhydrazyl (DPPH) Assay

As mentioned earlier, the DPPH test has been carried out (Mensor et al., 2001). The diluted sample with ethanol was mixed with an aqueous-ethanolic extract (30:70) from *J. mollissima* to make a final volume of 5 mL with different concentrations (4 mL). Then, for 40 min, this mixture was stored in the dark. The stated solution’s 517 nm absorbance was measured using a spectrophotometer. Each study was done three times, and the percentage of inhibition in ascorbic acid equivalency was measured. Equation below was used to compute the percentage of DPPH scavenging potential:

$$1\%=A\;\left(\text{blank}\right)\;–\;B\;\left(\text{sample}\right)\;/\;A\;\left(\text{blank}\right)\times \;100$$

#### 2.7.2 Measurement of nitric oxide (NOx) scavenging capacity

*J. mollissima* was extracted using an aqueous-ethanolic solution at a concentration of 10 mg/mL. Standard (Ascorbic acid) and extract were both serially diluted with distilled water to achieve concentrations of 1,000 and 2,000 μg/mL. Experiment solutions were stored at a constant 4°C. A freshly made Griess reagent was utilised in the process. To determine the optimal concentration of extract (1,000 and 2,000 g/mL), 0.5 mL of 10 mM sodium nitroprusside in phosphate-buffered saline was added to 1 mL of each extract concentration and incubated at 25°C for 3 h. The extract was spiked with a freshly made Griess reagent of the same volume. The extracts were left out of the control samples, but the volume of buffer used was kept constant. There were adequate quantities of extracts in the various hued tubes, but no sodium nitroprusside. A 96-well plate was loaded with 150 L of the reaction mixture. Based on our prior correspondence (Khan et al., 2021a; Khan et al., 2021b; Khan et al., 2022), we used a UV-Vis microplate reader (Alibaba, Hangzhou, China) to measure the absorbance at 546 nm. Using the following formula, we were able to determine the percentage of nitrite radical scavenging activity of extracts and ascorbic acid, as well as the percentage of inhibition by the standard.

$$\%\text{ nitrite radical scavenging activity}:A\;\left(\text{blank}\right)\;-\;B\;\left(\text{sample}\right)\;/\;A\;\left(\text{blank}\right)\times 100$$

#### 2.7.3 Peroxide radical scavenging assay

To conduct our experiments, we adapted a previously published method (Ruch, Cheng and Klaunig, 1989), preparing extract and standard solutions at two different concentrations (1,000 and 2,000 μg/mL), as well as a 43 mM H_2_O_2_ solution in a 0.1 M phosphate buffer (pH 7.4). After combining the sample solutions with the 3.4 mL of phosphate buffer, we added the H_2_O_2_ solution (43 mM) at a volume of 0.6 mL. A UV spectrophotometer was used to measure the absorbance at 230 nm. A sodium phosphate buffer devoid of hydrogen peroxide served as a blank. The following formula was used to determine the scavenging efficiency (in percentage terms) of H_2_O_2_:

$$\%\text{ Inhibition}:A\;\left(\text{blank}\right)\;-\;B\;\left(\text{sample}\right)\;/\;A\;\left(\text{blank}\right)\times 100$$

#### 2.7.4 Reducing power scavenging assay

The ability to reduce iron (III) is often used as a proxy for phenolic antioxidant activity (Ebrahimzadeh et al., 2010). The extracts’ reducing potential was determined using a method similar to that proposed by (Yen and Chen, 1995). Each extract was diluted in water to a concentration of between 1,000 and 2,000 μg/mL, then combined with potassium ferricyanide [K3Fe(CN)6] (2.5 mL, 1% concentration) and phosphate buffer (2.5 mL, 0.2 M, pH 6.6). The combination was simmered at 50°C for 20 min. Centrifuging at 3,000 rpm for 10 min and then adding 2.5 mL of 10% trichloroacetic acid interrupted the process. Two and a half millilitres of the upper solution layer were mixed with two and a half millilitres of distilled water and 0.5 mL of FeCl3 (0.1%) to determine the absorbance at 700 nm. The reaction mixture’s absorbance went up, showing that the reducing power had increased. Ascorbic acid was used as a good proxy for a positive control.

#### 2.7.5 Superoxide dismutase assay

Using a slightly altered version of the method reported by (Gülçin et al., 2004), the herb’s capacity to scavenge superoxide anion radicals was determined. The PMS, NADH, and NBT system was applied for the generation of superoxide radicals. We put 50 µL of the tested materials at different concentrations, along with 125 µL of NBT (300 M) and 125 µL of NADH (468 M) in a test tube containing 625 µL of Tris-HCl buffer (16 mM, pH 8.0). The reaction began after 125 µL (60 mM) of PMS was added to the mixture. Five minutes of room temperature incubation was followed by a vigorous vortexing of the mixture. And finally, the absorbance was measured with a spectrophotometer (Hitachi, U-1900 UV/VIS, Hitachi High-Technologies Corporation).

### 2.8 Anticancer activity

#### 2.8.1 Principle of MTT assay

The cell viability of plant extracts can be measured with a colorimetric assay using 3-(4,5-dimethythiazol-2-yl)-2,5-diphenyl tetrazolium bromide (MTT). Only viable cells with active mitochondrial enzymes can be utilized in this method to determine the cytotoxicity of a plant extract. The mitochondrial enzyme mitochondrial reductase contributes to this method. The formation of formazan causes the yellow MTT reagents to change hue to a deep blue during the reaction. Toxicity to cells, disruption of metabolic maturation, and a diminished bioassay presence are all components of this complex. Mitochondrial reductase is the enzyme that converts the yellow MTT reagent into the purple formazan (Mosmann, 1983). Afterward, the formazan was mixed with the DMSO and the ethanol. The MTT assay was chosen as the cell viability assay due to its low cost and rapid turnaround time.

##### 2.8.1.1 Diluting herbal extracts

The plant extract was dissolved in DMSO or water depending on the user’s preference. Every one of the 100 mM stock solutions was frozen at −20°C until use. After being combined with a DMEM medium, the pH levels of these solutions were found to be between 7 and 8.

##### 2.8.1.2 Protocol for the MTT assay

The cells were seeded at a density of 1 × 10^5^ cells/mL in 100 ul complete growth medium in a 96-well plate and incubated for 24 h at 37°C and 5% CO_2_. 100 ul of 1:20 diluted extracts were used to treat the cells at concentrations of 0 (Vehicle control), 100, 200, 300, and 400 ug/mL after incubation at 37°C with 5% carbon dioxide for 4, 24, and 48 h. After 24 h, the 96-well plates were foil-wrapped to maintain the MTT’s sensitivity to light, and 10 ul of yellow MTT reagent from a 5 mg/mL stock solution was added to each well. After that, the plates were incubated for another two to 4 hours at 37°C and 5% carbon dioxide. The precipitated crystals were entirely dissolved in 100ul of DMSO. To determine the absorbance of the samples in the microplates, a microplate reader (Tecan Infinite^®^ M200) was used. There were three independent trials of the experiments done.

### 2.9 Cell morphology analysis

Under an inverted and phase contrast microscope, we observed morphological changes in HepG2 cells treated with 25 ug/mL of *Jm* after 24, 48, and 72 h of incubation (Leica, Wetzlar, Germany).

### 2.10 Ultra morphological analysis of apoptosis with acridine orange (AO)/propidium iodide (PI) staining

Fluorescence microscopy was used for analysis after standard protocols for viewing cells with AO and PI had been performed. (Leica coupled with Q-Floro Software; Leica Microsystems, Wetzlar, Germany). About 1 × 10^6^ cells/mL were seeded into a 25 mL culture flask, and the cells were subsequently treated to 6 g/mL of *Jm* for 48 h. The cells were centrifuged at 200 g for 5 min to remove the medium. Before being stained with AO/PI, the cells were given two cold washes in phosphate-buffered saline (PBS). The cells on the glass slide were then treated with freshly produced AO/PI at a 10 g/mL concentration. The slides were analyzed around 30 min before the fluorescence faded by using fluorescence microscopy.

### 2.11 Annexin V and propidium iodide staining

Six-well microtiter plates were used to cultivate the cells (at a concentration of 10^5^–10^6^ cells/mL) in serum-free conditions for 24 h. The following 24, 48, and 72 h, they were administered escalating doses of *Jm*. Annexin V/propidium iodide (PI) staining was used to distinguish between apoptotic, late apoptotic, and non-apoptotic cells at the specified timeframes. To stain for PI and annexin V-FITC, cells were washed twice in ice-cold PBS and then suspended in 100 L of Annexin V binding buffer (0.1 MHepes/NaOH (pH 7.4), 1.4M NaCl, 25 mM CaCl2) for 15 min. FACS analysis was performed on a FACS Calibur flow cytometer using Cell Quest software (BD Biosciences, San Jose, CA, United States) to ascertain cell viability.

### 2.12 Collection of metabolic data and blood sample

Urine samples were taken from rats on days 0 and 10, and 21 while they were housed in metabolic cages with free access to running water. Urine volume and water consumption were recorded. The sodium and potassium concentrations in the urine and the urine production rate were measured.

Blood samples were also taken on the 0, 10th and 21st days to measure CK-MB, LDH, Troponin I, serum sodium, and potassium level by collecting blood by the retro-orbital method.

On day 21, after fasting for 12 h, we weighed all the animals and put them to sleep with a mixture of xylazine and ketamine (1:10). Heart puncture was used to draw blood and clot it for testing. Centrifugation at 3,000 rpm for 15 min was used to separate the serum.

We washed the heart in normal saline to get rid of the blood, then put it in formalin for later histopathological examination.

### 2.13 Biochemical parameters in serum for cardiotoxicity

Serum concentrations of creatinine kinase-MB (CK-MB), Troponin-I, and lactate dehydrogenase (LDH) were estimated using commercially available standard enzymatic kits. Serum electrolytes like serum sodium and serum potassium were also measured.

#### 2.13.1 Calculation of CK-MB

CK-MB (Creatine kinase myocardial band) is a very sensitive cardiac biomarker used to detect any cardiac damage due to any toxic drug or myocardial infarction.

CK-MB assay kits are used for determining its values in the blood. SBio assay kit measured CK-MB levels in the animals intoxicated by doxorubicin.

A blood sample was collected at 0, 10th^,^ and 21st day and allowed to stand for 20 min. Then, it was centrifuged to obtain serum and analyzed using a UV spectrophotometer at 340 nm using the following procedure.

The assay required 1 mL of working reagent, which was heated to 37 °C for 1 min, followed by adding 0.05 mL of sample. After 10 min of blending, check the initial absorbance (A0) and take additional readings at 1-, 2-, and 3-min intervals. Please use the following formula to determine the average absorbance rate of change (ΔA/min)

$$\text{CK}‐\text{MB}\text{activity}\text{in}U/L37^{0}C=\Delta A/\min.\;\times \;6666$$

#### 2.13.2 Measurement of LDH in serum

LDH (Lactate dehydrogenase) was measured using a Human kit in which a blood sample was collected at 0, 10th, and 21st day and allowed to clot for 20 min. Then, this clotted blood was centrifuged in the centrifugation machine for 15 min at 3,000 rpm. The serum layer was separated from the blood and was collected. The serum was analyzed in a UV spectrophotometer at 340 nm using the following procedure.

The average absorbance variation per minute (ΔA/min) is used to derive absorbance readings. Multiplying the sample’s ΔA/min by 8,095 yields a measure of LDH activity.

$$U/l\;\left(25^{0},30^{0}\right)=\Delta \;A/\min.\;X$$

where X is the LDH value and is measured in triplicate.

#### 2.13.3 Estimation of serum potassium and sodium levels

Electrolyte profiles screen electrolytes and acid-base imbalances and monitor treatment efficacy. When diagnosing a medical issue, electrolytes like sodium, potassium, chloride, and bicarbonate may be indicators (Iqbal and Yahya, 2021; Omer Iqbal et al., 2021).

Serum samples were analyzed using a flame photometer to determine sodium and potassium concentrations (Sherwood Model 410, United Kingdom).

Samples were diluted 1:200 for the measurement of sodium and potassium in serum; The entire process was carried out twice to ensure its integrity.

### 2.14 Statistical analysis of data

Data analysis and an indication of statistical significance between groups in an experiment were conducted using analysis of variance (ANOVA) using one-way analytical variance and Bonferroni’s all-mean *post hoc* test. To analyze the information, Graph Pad Prism was used. Results were deemed to be statistically significant when the *p*-value was less than 0.05.

## 3 Results

This research was carried out to ascertain whether or not *J. mollissima* may mitigate the cardiotoxicity and nephrotoxicity caused by doxorubicin. 70% aqueous ethanolic extract of *J. mollissima* (*Jm*) was prepared and used for experimental purposes.

### 3.1 Preliminary phytochemical evaluation and antioxidant assays

A phytochemical analysis of *J. mollissima* hydroalcoholic extract revealed a rich profile of bioactive components (Table 1). In a number of different antioxidant studies, the 1,000 g/mL concentration of *Jm* hydroalcoholic extract showed the greatest DPPH level (87.80%) and the highest reducing power (73.24%); NO (Nitric oxide), H_2_O_2_ (hydrogen peroxide), and SOD (superoxide dismutase) showed 92.12, 89.86, and 80.40% inhibition, respectively. Table 2 lists some of the many tests that have been used to assess antioxidant activity.

**TABLE 1:** Phytoconstituents present in aqueous-ethanolic extract of the *Jatropha mollissima*.

Serial number	Test	Ethanolic extract
1	Alkaloid	Positive
2	Saponins	Positive
3	Tannins	Positive
4	Anthraquinones	Positive
5	Coumarins	Positive
6	Phenols	Positive
7	Flavonoids	Positive

• Ppt, Precipitates; cm, Centimeter.

**TABLE 2:** Results from multiple assays examining *Jatropha mollissima* plant extract for antioxidant activity.

Concentration (μg/mL)	Extract inhibition					Standard inhibition				
	DPPH	Reducing power	NO	H_2_O_2_	SOD	DPPH	Reducing power	NO	H_2_O_2_	SOD
1,000	87.80	73.24	92.12	89.86	80.40	97.25	81.27	92.84	96.01	95.25
2,000	94.50	86.02	94.05	91.78	85.63	96.14	88.15	88.32	91.25	93.26

### 3.2 Protective effect of *Jatropha mollissima* (*Jm*) against doxorubicin-induced cardiotoxicity and nephrotoxicity

Doxorubicin is an anthracycline antibiotic used for various types of cancers like breast, lung, gastric, ovarian cancer, pediatric cancers, hematologic malignancies, and soft tissue sarcomas, Wilms’ tumor, neuroblastoma, bone sarcomas, transitional cell bladder carcinoma, bronchogenic carcinoma, and thyroid carcinoma. The use of anthracyclines is limited due to their toxic effects. Several mechanisms are proposed through which it causes toxicity. It may generate free radical formation by NADPH-dependent reductases, which form anthracycline semiquinone free radicals by reducing anthracyclines, causing apoptosis in cardiac cells (Kirstein, 2009; Ghigo et al., 2016).

Doxorubicin (Doxo) interacts with mitochondrial enzymes to generate reactive oxygen species (ROS), which in turn cause lipid peroxidation and DNA damage; DNA binding; DNA alkylation; inhibition of DNA unwinding, strand separation, and helicase activity; inhibition of enzyme topoisomerase II, which results in DNA damage and apoptosis; and membrane damage; in the presence of iron, doxorubicin forms a complex with it to bind with DNA to generate reduced They are not only toxic to the heart but also to the kidneys and liver (Outomuro et al., 2007; Salazar-Mendiguchía et al., 2014).

#### 3.2.1 Effect of *Jm* on body weights of animals

**FIGURE 1:**
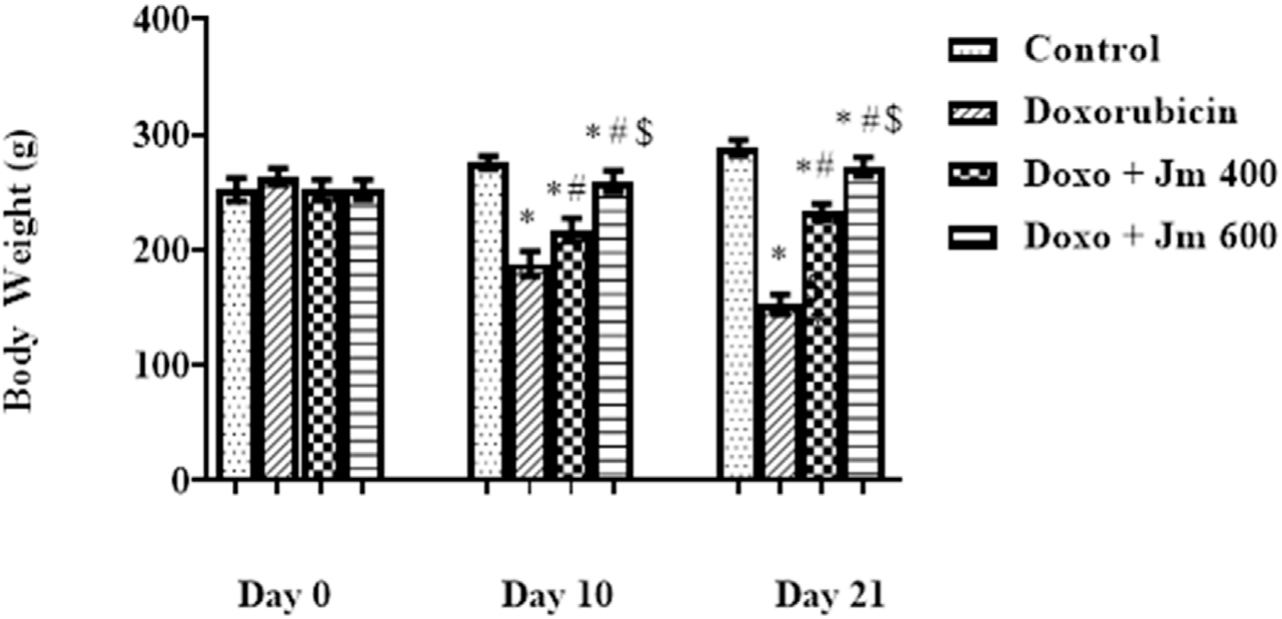
Body weight of normal control, doxorubicin, Dox + *Jm* 400, and Dox + *Jm* 600. Results are deemed to be statistically significant (*) if *p* is less than 0.005. For each day, a * denotes statistical significance when compared to the control group, # to Doxorubicin, and $ to Dox + *Jm* 400.

The weight of the animals was recorded on days 0 and 10th, and 21st. During the 21-day experiment, the animals received intraperitoneal injections of 10 mg/kg of doxorubicin on day 1. The groups had no statistically significant difference in day 0 body weight (all *p* > 0.05). Doxorubicin-treated rats lost weight throughout the study compared to their weight on day zero (all *p* < 0.05). Weight increases were observed across all *Jm*-treated groups, particularly those given 400 and 600 mg/kg (all *p* < 0.05) as shown in Figure 1.

In conclusion, both the control and *Jm* groups of rats had a rise in body weight. Contrary to this, Dox-treated groups showed a decreasing body weight pattern due to decreased appetite and less food intake. In contrast, body weight was significantly increased in animals treated with *Jm* intoxicated with Doxo.

#### 3.2.2 Heart weight

Heart weight was found to be considerably lower in doxorubicin-treated rats relative to the control group. Co-administered different doses of *J. mollissima* extracts, i.e., 400 and 600 mg/kg with doxorubicin, caused a slight increase in heart weight, respectively as shown in Table 3.

**TABLE 3:** The role of *Jm* in Doxo-induced cardiac weight alterations.

Groups	Heart weight (g)
Normal control	0.83 ± 0.46
Doxorubicin	0.56 ± 0.42 (*)
Doxo + *Jm* 400 mg/kg	0.59 ± 0.47 (#)
Doxo + *Jm* 600 mg/kg	0.65 ± 0.48 ($)

Results are deemed statistically significant (*) if *p* < 0.005. For each day, a * denotes statistical significance compared to the control group, # to Doxorubicin, and $ to Dox + *Jm* 400.

### 3.3 Effect of *Jm* on cardiac marker enzymes

#### 3.3.1 Effect of *Jm* on serum creatine kinase (CK-MB)

Creatine kinase is categorized into three types: CK-BB, CK-MB, and CK- form, which is specific for the brain, heart, and muscle form, respectively. The heart is sensitive to the reactive oxygen species due to fewer anti-oxidant enzymes like superoxide dismutase, catalase, and glutathione, which are protective enzymes in the heart. Cardiolipin is a phospholipid found in the mitochondrial membrane of heart cells, and doxorubicin binds to it quite strongly (El-Sayed et al., 2011).

**FIGURE 2:**
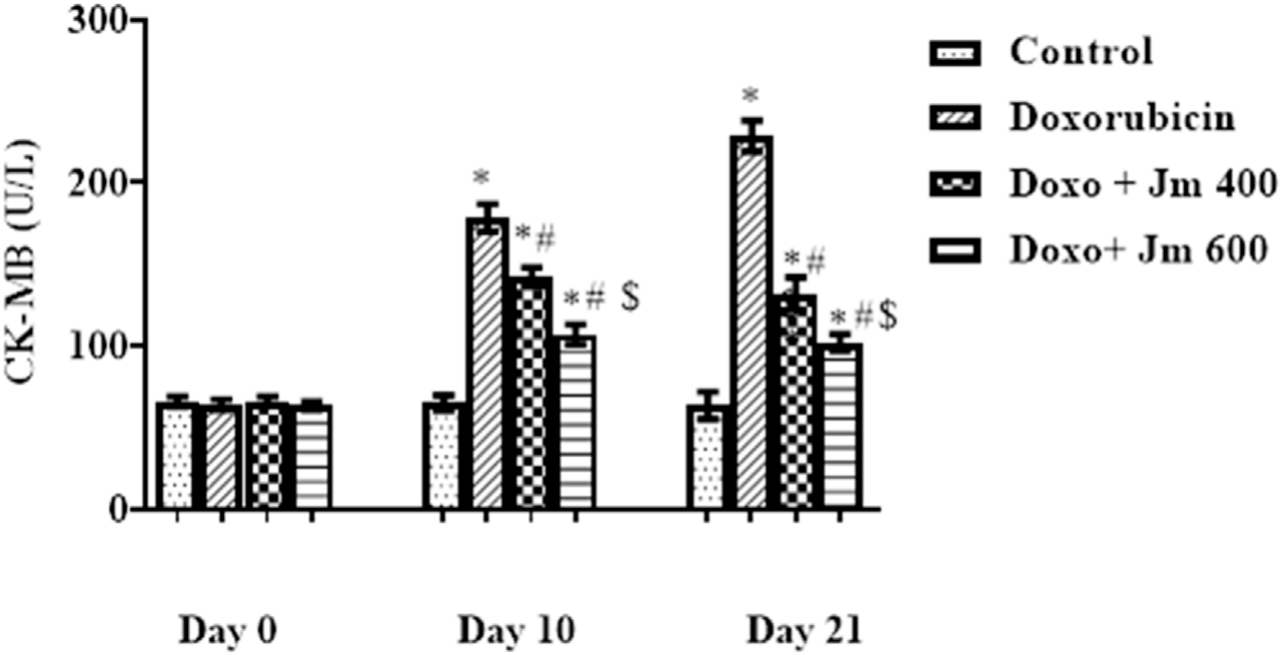
Serum CK-MB levels of normal control, doxorubicin, Dox + *Jm* 400, and Dox + *Jm* 600. Results are deemed to be statistically significant (*) if *p* is less than 0.005. For each day, a * denotes statistical significance when compared to the control group, # to Doxorubicin, and $ to Dox + *Jm* 400.

Serum levels of cardiac marker enzyme CK-MB increased dramatically in Doxorubicin-treated rats compared to control (all *p* < 0.05) 21 days after administration of a single bolus dose of doxorubicin (10 mg/kg) (all *p* > 0.05). Serum levels decreased (all *p* < 0.05) in response to *Jm* administration in the 400 mg/kg and 600 mg/kg treatment groups, as depicted in the Figure 2 below.

#### 3.3.2 Effect of *Jm* on serum lactate dehydrogenase (LDH)

Lactate dehydrogenase is a biomarker for heart muscle damage present in two isoforms, i.e., H form is specified for the heart while M is specific for muscles. Increased level of LDH indicates heart damage. It is a quantitative index of cell integrity (Giri et al., 2004).

**FIGURE 3:**
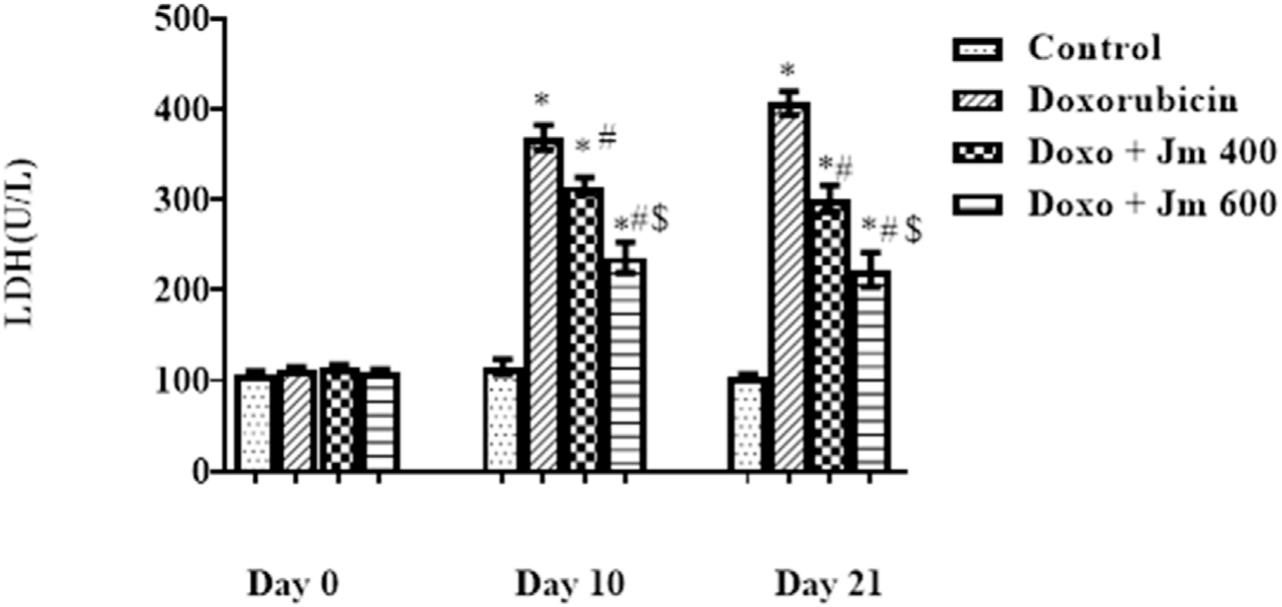
Serum LDH levels of normal control, doxorubicin, Dox + *Jm* 400 and Dox + *Jm* 600. Results are deemed to be statistically significant (*) if *p* is less than 0.005. For each day, * denotes statistical significance when compared to the control group, # to Doxorubicin, and $ to Dox + *Jm* 400.

Serum levels of LDH were increased dramatically in Doxorubicin-treated rats compared to control (all *p* < 0.05) 21 days after administration of a single bolus dose of doxorubicin (10 mg/kg) (all *p* > 0.05). Serum levels of LDH are decreased (all *p* < 0.05) in response to *Jm* administration in both the 400 mg/kg and 600 mg/kg treatment groups, as depicted in the Figure 3 below.

#### 3.3.3 Effect of *Jm* on troponin I (cTnI)

Troponin is a very sensitive parameter to detect any myocardial damage. It comprises three proteins—troponin I, C, and T—along the muscle’s thin filament. Troponin I provides a longer duration for detecting myocardial injury, i.e., 6–10 days, compared to troponin C and T, which exits only for 4–6 h (Rashid et al., 2013).

**FIGURE 4:**
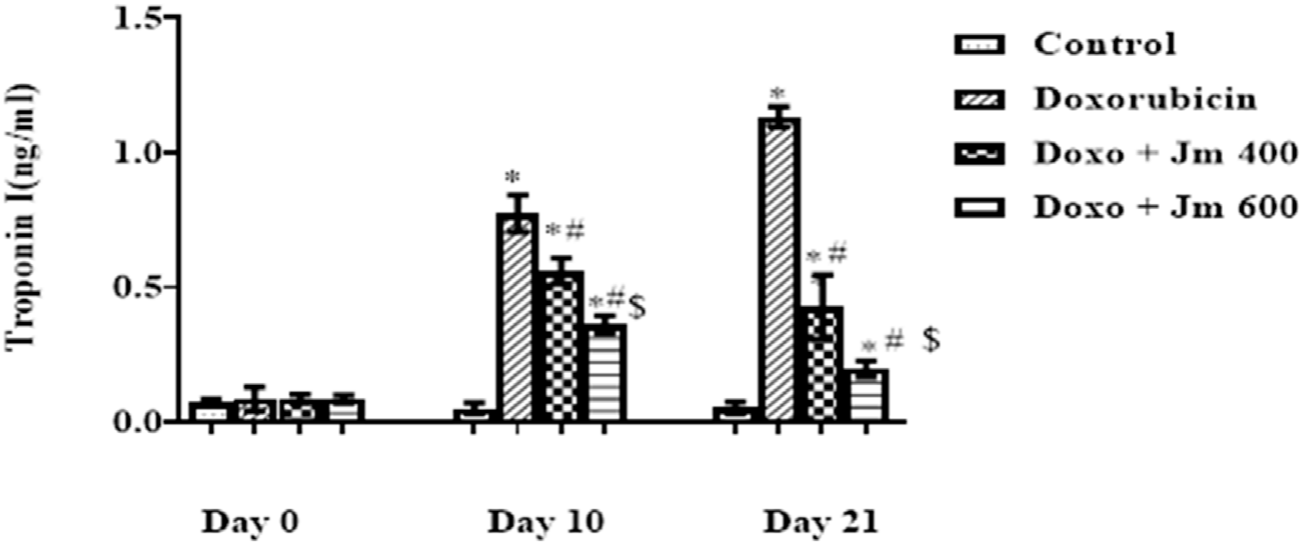
Serum Troponin I (cTnI) levels of normal control, doxorubicin, Dox + *Jm* 400 and Dox + *Jm* 600. Results are deemed to be statistically significant (*) if *p* is less than 0.005. For each day, a * denotes statistical significance when compared to the control group, # to Doxorubicin, and $ to Dox + *Jm* 400.

Levels of Troponin I were increased dramatically in Doxorubicin-treated rats compared to control (all *p* < 0.05) 21 days after administration of a single bolus dose of doxorubicin (10 mg/kg) (all *p* > 0.05). Troponin I (cTnI) levels are decreased (all *p* < 0.05) in response to *Jm* administration in both the 400 mg/kg and 600 mg/kg treatment groups, as depicted in the Figure 4 below.

#### 3.3.4 The implication of *Jm* in sodium concentration in blood

**FIGURE 5:**
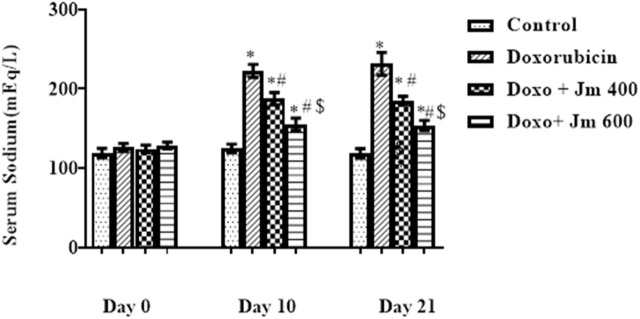
Serum sodium levels of normal control, doxorubicin, Dox + *Jm* 400, and Dox + *Jm* 600. Results are deemed to be statistically significant (*) if *p* is less than 0.005. For each day, a * denotes statistical significance when compared to the control group, # to Doxorubicin, and $ to Dox + *Jm* 400.

The sodium concentration was determined by centrifuging blood at 3,000 rpm for 15 min to separate serum. The sample was diluted 1:200 and examined by a flame photometer. Increases in serum sodium were seen in the doxorubicin-treated group compared to the control group after a 10 mg/kg dosage on day 1. A small but statistically significant sodium decrease between groups gave 400 and 600 mg kg of *Jm* (all *p* < 0.05) as shown in Figure 5.

#### 3.3.5 The implication of *Jm* in potassium concentration in blood

**FIGURE 6:**
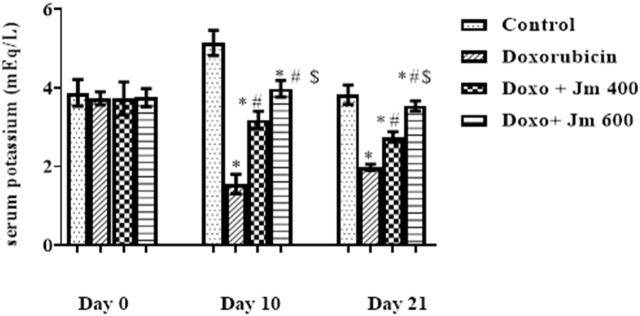
Serum potassium levels of normal control, doxorubicin, Dox + *Jm* 400, and Dox + *Jm* 600. Results are deemed to be statistically significant (*) if *p* is less than 0.005. For each day, a * denotes statistical significance when compared to the control group, # to Doxorubicin, and $ to Dox + *Jm* 400.

The potassium concentration was determined by centrifuging blood at 3,000 rpm for 15 min to separate serum. It was then analyzed by using a flame photometer. Serum potassium levels were lower in the group that received a single dose of doxorubicin (10 mg/kg) on day one compared to the normal control group (all *p* > 0.05). In contrast, there is a modest rise in sodium levels in groups given *Jm* at doses of, i.e., 400 and 600 mg kg (all *p* < 0.05), as depicted in Figure 6.

### 3.4 Histopathology

**FIGURE 7:**
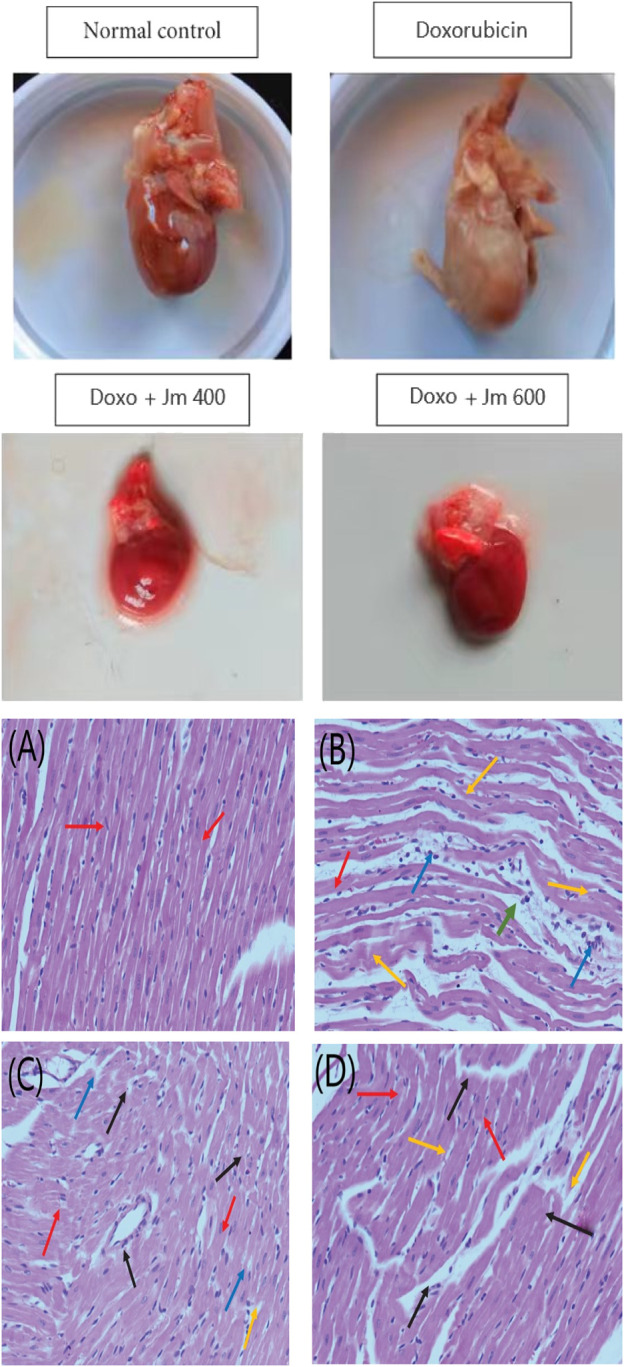
Histopathological section of the heart; **(A)** Control group **(B)** Doxorubicin (10 mg/kg) **(C)** Treatment group Doxo + *Jm* 400 mg/kg **(D)** Treatment group (Doxo + *Jm* 600 mg/kg) in doxorubicin-induced cardiotoxicity. The red arrow represents a healthy cardiomyofibril; the green arrow demonstrates perivascular cuffing of the vasa vasorum, which is associated with intimal fibrosis; the orange arrow demonstrates myocytolysis; and the blue arrow demonstrates myonecrosis.

Hearts from treated and untreated rats were histologically examined to detect lesions and damage caused by doxorubicin. And to see if *J. mollissima* extract can prevent the heart damage caused by doxorubicin as shown in Figure 7.

### 3.5 *Jatropha mollissima* protective action against the kidney damage caused by doxorubicin

#### 3.5.1 Kidney weight

In animals given doxorubicin, kidney weight increased dramatically compared to controls. Combining extract and doxorubicin led to a moderate decrease in kidney mass as shown in Table 4.

**TABLE 4:** Effect of *Jm* on Doxorubicin-induced changes in kidney weight.

Groups	Weight of the kidneys
Normal control	3.9 ± 0.21
Doxorubicin	5.2 ± 0.32 (*)
Doxo + *Jm* 400 mg/kg	4.7 ± 0.27 (#)
Doxo + *Jm* 600 mg/kg	4.00 ± 0.26 ($)

Results are deemed statistically significant (*) if *p* < 0.005. For each day, a * denotes statistical significance compared to the control group, # to Doxorubicin, and $ to Dox + *Jm* 400.

#### 3.5.2 Effect of *Jm* hydroalcoholic extract on urine output

**FIGURE 8:**
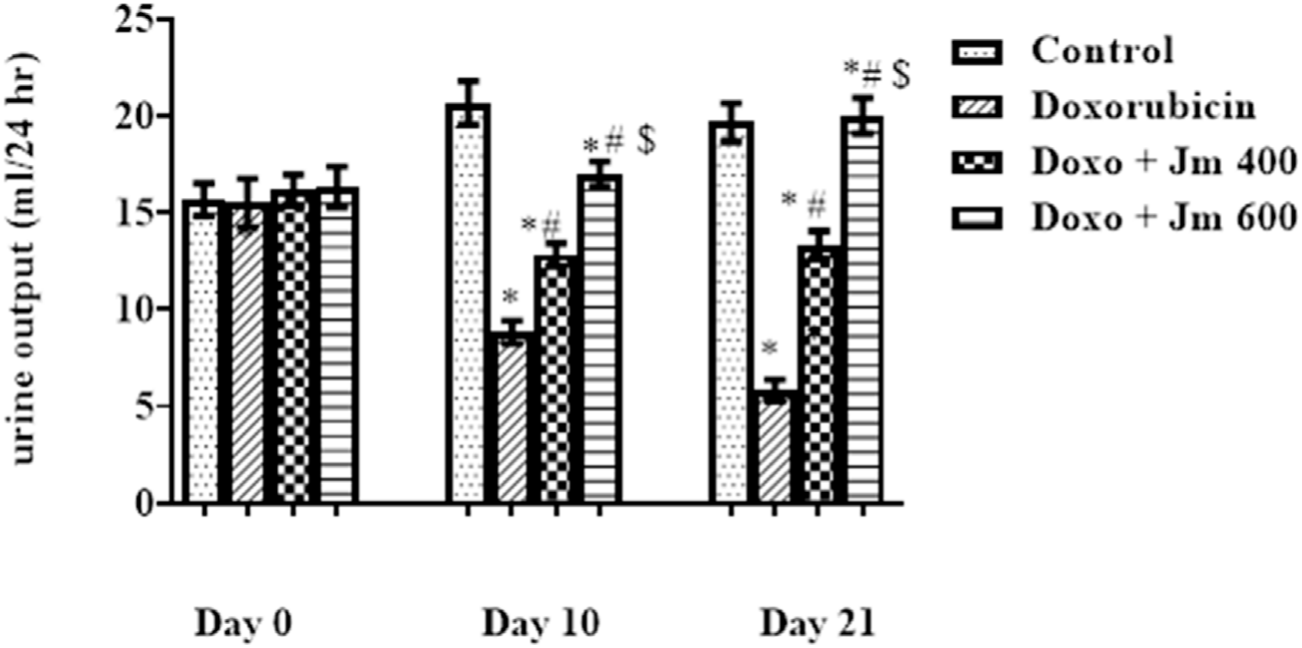
Urine output of normal control, doxorubicin, Dox + *Jm* 400 and Dox + *Jm* 600. The results are considered significant (*) if *p* < 0.005. * indicates *p* < 0.05 vs. normal control, # indicates *p* < 0.05 vs. Doxorubicin, $ indicates *p* < 0.05 vs. Dox + *Jm* 400 on respective days.

Rats were placed in metabolic cages for 24 h with access to running water but no food to determine this. After 24 h, the urine volume was measured, and it was shown that the Doxorubicin intoxicated group had much lower urine volume and a darker color than the normal control group. Figure 8 shows that as the study progresses, the *Jm*-treated and the Doxo-intoxicated groups produced more urine when given *Jm* at 400 and 600 mg kg (all *p* < 0.005).

#### 3.5.3 Effect of *Jm* hydroalcoholic extract on urinary sodium levels

**FIGURE 9:**
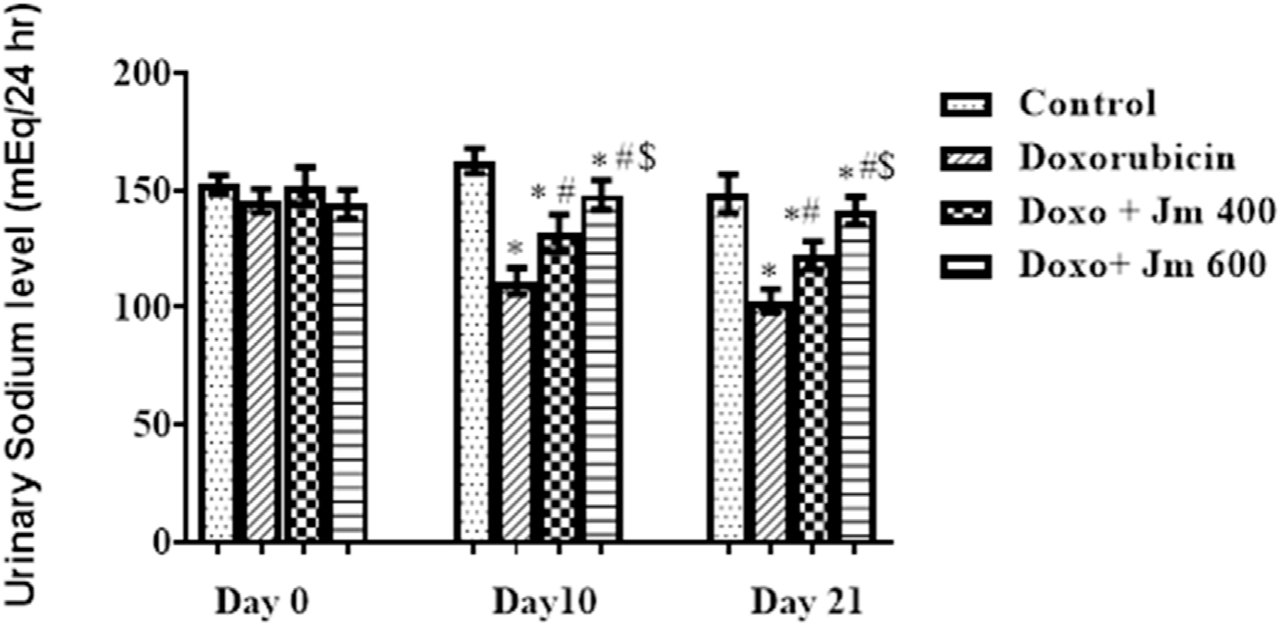
Urinary sodium levels of normal control, doxorubicin, Dox + *Jm* 400 and Dox + *Jm* 600. The results are considered significant (*) if *p* < 0.005. * indicates *p* < 0.05 vs. normal control, # indicates *p* < 0.05 vs. Doxorubicin, $ indicates *p* < 0.05 vs. Dox + *Jm* 400 on respective days.

For 24 h, rats were given free access to running water and kept in metabolic cages but no food to determine this. After 24 h, urinary sodium was measured, and it was shown that the Doxorubicin intoxicated group had much lower levels of sodium in the urine and a darker color than the normal control group. The Figure 9 shows that as the study progresses, both the *Jm*-treated and the Doxo-intoxicated groups increase the sodium level in urine when given *Jm* at doses of 400 and 600 mg kg (all *p* < 0.005).

### 3.6 Histopathology of kidney

**FIGURE 10:**
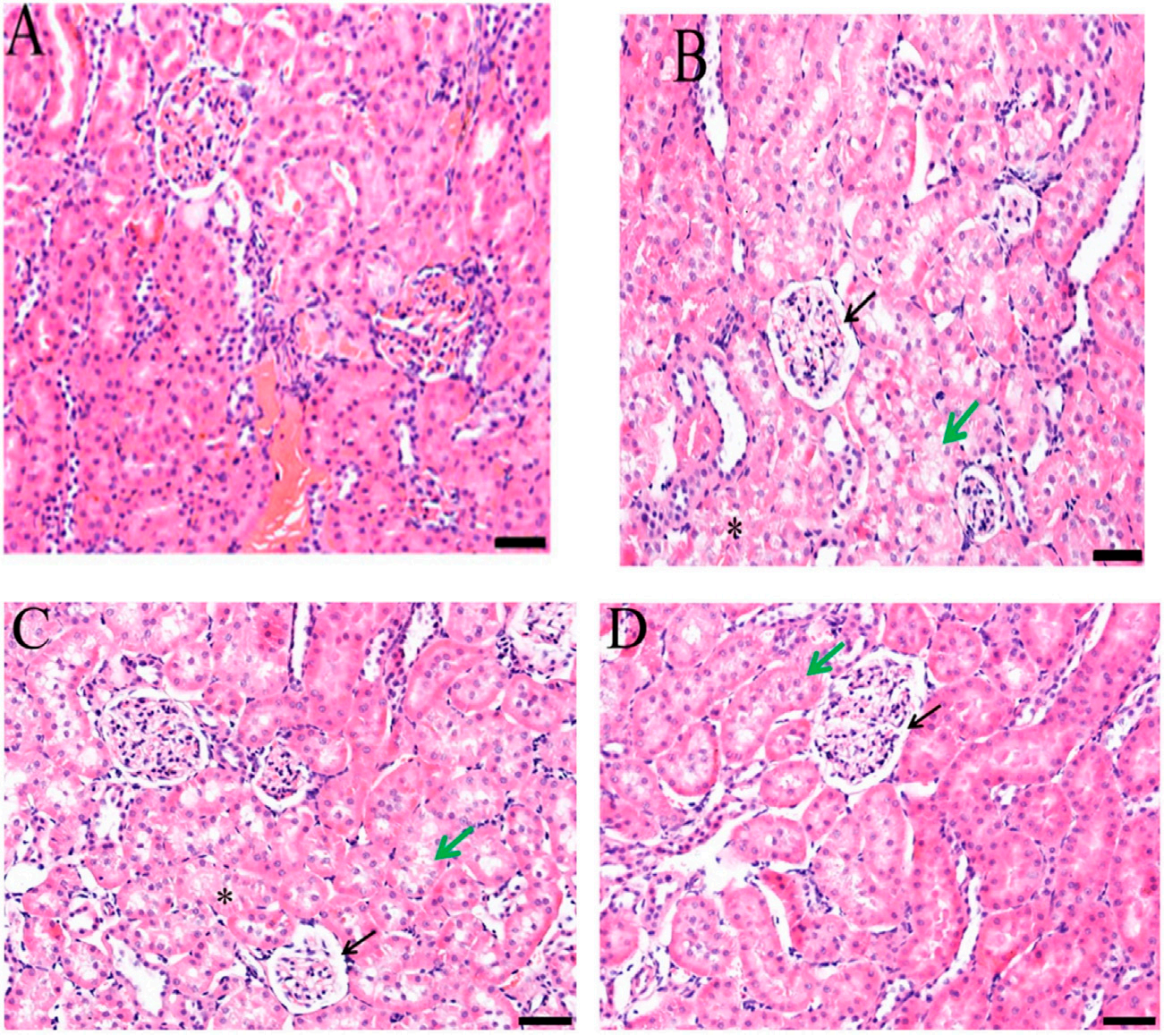
Histopathological section of the kidney; **(A)** Control group **(B)** Doxorubicin **(C)** Treatment group (Dox + *Jm* 400 mg/kg) **(D)** Treatment group (Dox + *Jm* 400 mg/kg) in doxorubicin-induced nephrotoxicity. Necrosis atropy detected in epithelial cells of proximal and distal tubule (*); Green arrow indicates widespread oedema, vacuolation, and hydrophic degeneration; Black arrow denotes atrophic glomerulus and extensive capsular space.

Doxorubicin causes nephrotoxicity, which was confirmed by histopathology as lesions were formed in contrast to the control group. Improvement was observed in slides of groups treated with *Jm* at 400 and 600 mg/kg doses as shown in Figure 10.

### 3.7 Determination of cell viability by MTT assay

**FIGURE 11:**
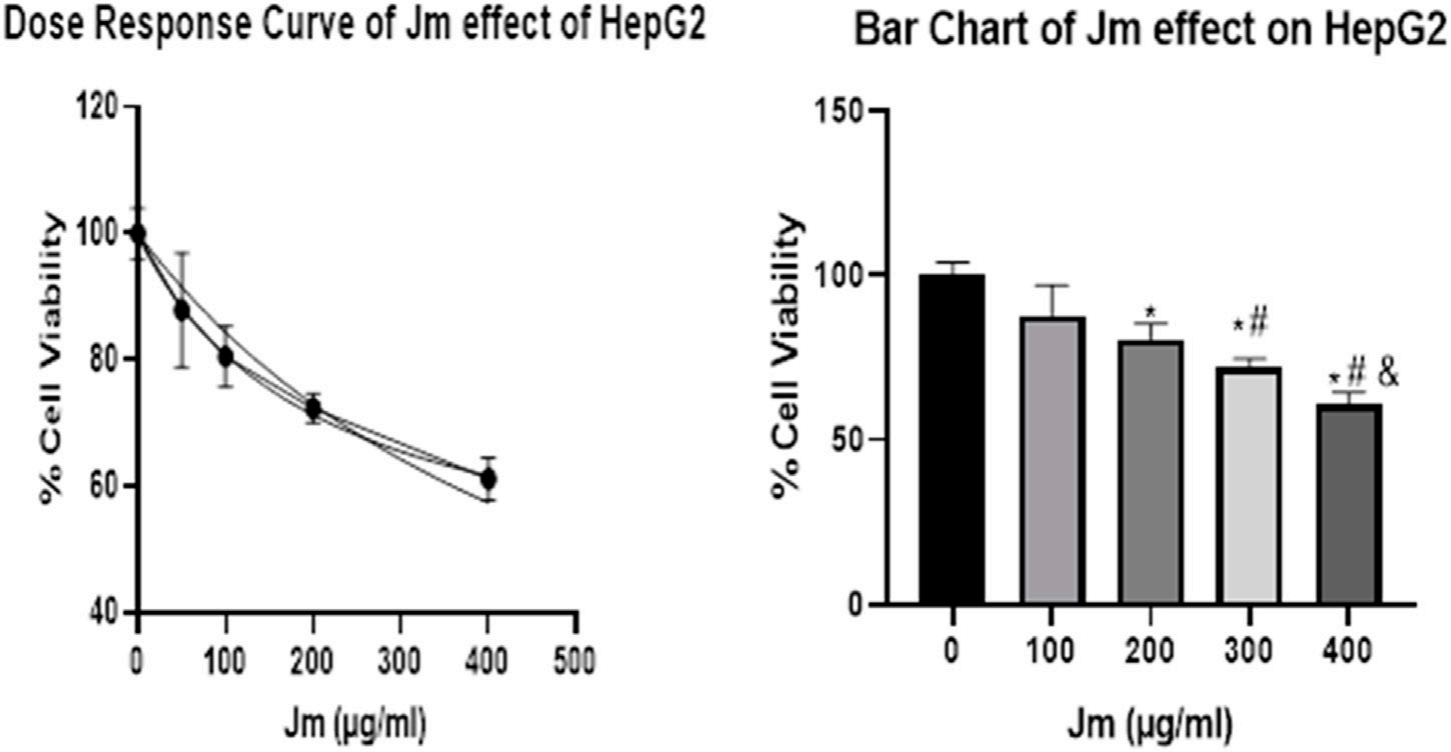
Cell Viability Assay. *Jatropha mollissima* extracts on the HepG2 cell line as a percentage of cell viability by MTT assay **(A)**. *Jm*’s effect on HepG2 as a function of dose **(B)** Bar chart of *Jm* effect on HepG2 results 24 h after treatment at various doses on HepG2. One-way ANOVA was used to make the comparisons between the samples. The results were reported as the mean standard error of the mean (*n* = 3), with triplicates of each experiment included. **p < 0.01, ***p < 0.001, and ****p < 0.0001 versus the control group.

The hepatocellular carcinoma (HepG2) cell line also showed a concentration-dependent response to the plant extracts. The highest concentration of plant extracts (400 g/mL) was shown to inhibit cells to a greater extent than any of the lower doses tested. As shown in the Figure 11, the percentage of cells inhibited rose with the concentration of the extracts. However, the effect on cell proliferation was minimal after 24 h of treatment with doses up to 90 g/mL of crude ethanolic extract in HepG2 cells. Cell viability declined by over 50% at doses higher than 100 μg/mL (Figure 11). Cell viability decreased proportionally after treatment with a different concentration of *Jm* extract. However, as Figure demonstrates, safe levels for healthy cells with a 90% or above viability are below 90 or 100 μg/mL. This study showed that *Jm*’s crude ethanolic extract was safe at modest dosages in animal models, while it cautioned against using high doses for an extended period.

### 3.8 Morphological alterations in the HepG2 cell line caused by *Jm*

**FIGURE 12:**
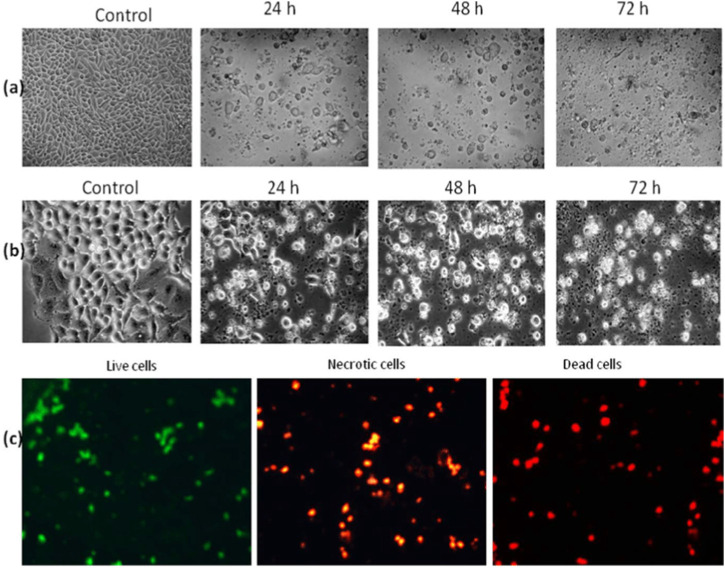
Morphological observation: **(A)** Inverted microscopic observation of HepG2 **(B)** Phase contrast imaging of HepG2, © AO/PI staining-based fluorescent analysis.

Using inverted and phase contrast microscopy, this study compared the morphology of untreated HepG2 hepatocellular carcinoma cell lines to that of HepG2 cells treated with active extracts of *J. mollissima* after 24, 48, and 72 h. Apoptosis was observed in the treated cells, as evidenced by the production of apoptotic bodies, membrane blebbing, nuclear condensation, and cell shrinkage (as depicted in the Figure 12). The results were stunning when comparing the total number of treated cells to that of untreated cells.

### 3.9 Morphological alteration in HepG2 cells by *Jm* by acridine orange (AO)/propidium iodide (PI) staining

The cells were examined in a customary way using AO and PI, and the results were analyzed using fluorescence microscopy. (Leica coupled with Q-Floro Software; Leica Microsystems, Wetzlar, Germany). According to our findings, viable cells were identified as having intact nuclei and being stained with green fluorescence. Apoptotic cells may be distinguished by the orange coloration caused by AO binding to denatured DNA, which revealed nuclear condensation, fragmentation, apoptotic bodies, and cytoplasmic blebbing. Additionally, the binding of AO to denatured DNA results in a crimson color, indicating dead or necrotic cells.

### 3.10 Staining using annexin V-FITC and propidium iodide

**FIGURE 13:**
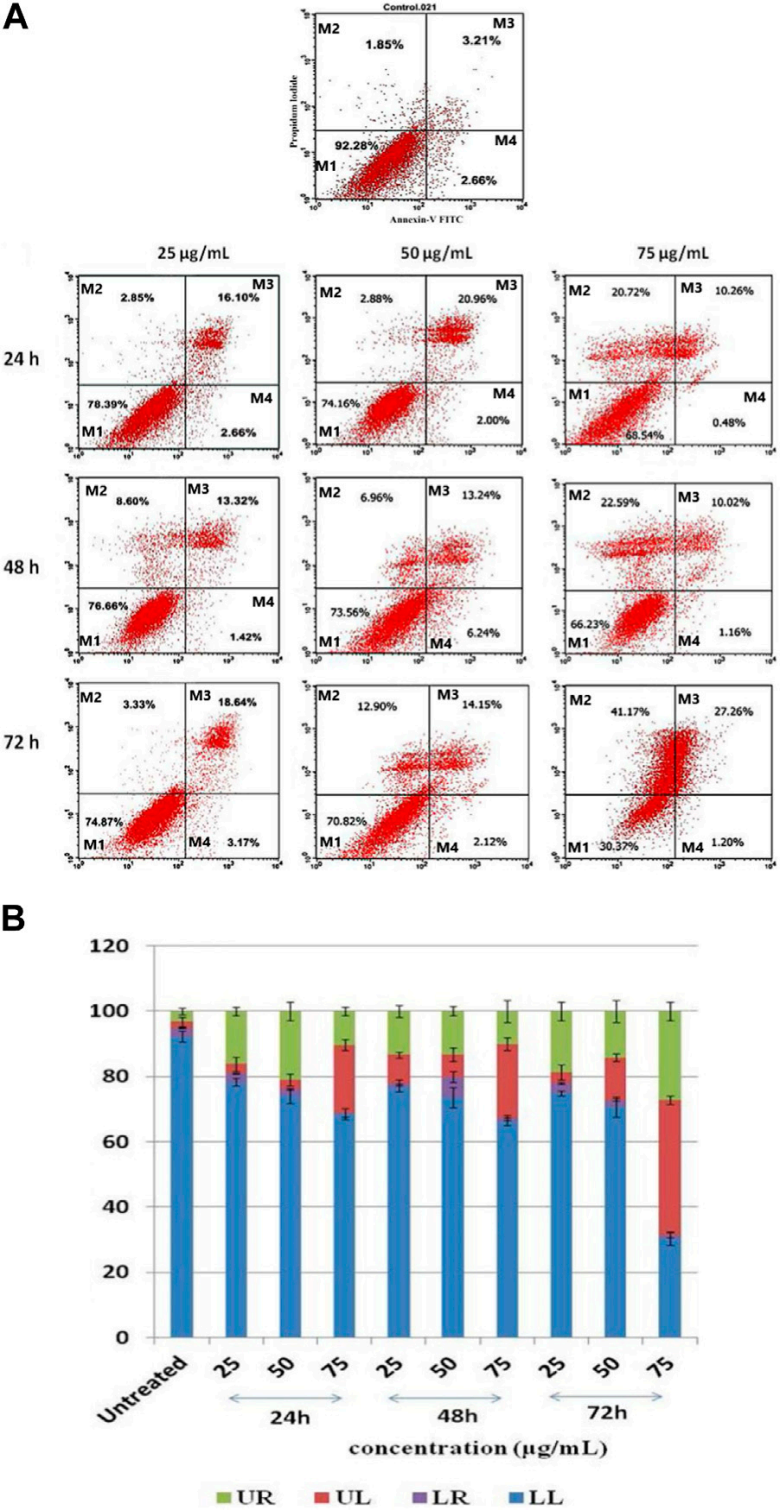
**(A)** *Jatropha mollissima* extract-induced apoptosis in HepG2 cancer cells as examined by Annexin V-FITC/PI. 25, 50, and 75 g/mL are the corresponding concentrations of *Jm*. The durations of *Jm* incubation of the cells were 24, 48, and 72 h. M1 represents viable cells, M2 represents primary necrotic cells, M3 presents late apoptotic cells, and M4 presents apoptosis. The results were reported as the mean standard error of the mean (*n* = 3), with triplicates of each experiment included. **p < 0.01, ***p < 0.001, and ****p < 0.0001 versus the control group. **(B)** A histogram showing the percentages of HepG2 cells that have undergone late apoptosis (UR), primary necrosis (UL), apoptosis (LR), and viable cells (LL).

When HepG2 cells are exposed to *J. mollissima*, a greater proportion enter the early apoptotic phase (as indicated in Figure 13 below) extract compared to untreated cells (*p* < 0.05). From these results, it can be concluded that *Jm* can significantly and dose-dependently trigger apoptosis in Hep G2 cells. One of the six diagnostic features of cancer is the avoidance of apoptosis. Additional research on the possible mechanism is required in the future.

## 4 Discussion

Doxorubicin, an anthracycline antibiotic, is useful in treating a wide variety of cancers, such as breast, lung, gastric, ovarian cancer, pediatric cancers, hematologic malignancies, and soft tissue sarcomas, Wilms’ tumor, neuroblastoma, bone sarcomas. However, it has limited use due to severe toxic side effects, including cardiotoxicity, which may lead to cardiomyopathy and heart failure depending on the dose of doxorubicin from acute or chronic drug harmful effects (Outomuro et al., 2007; Salazar-Mendiguchía et al., 2014).

There is a worldwide trend towards natural remedies to cure different diseases as they play an important role in many cardiac diseases with minimal or no side effects. *Jatropha mollissima* has an anti-oxidant potential as it possesses free radical scavenging potential. The ability of *J. mollissima* extract to suppress superoxide and hydroxyl free radicals is dose-dependent. Additionally, it dose-dependently inhibits lipid peroxidation and protein oxidation (M. O. Iqbal and Yahya, 2021). They allow the body to recover its normal levels of antioxidant enzymes like catalase, superoxide dismutase, and peroxidases that shield the body from toxic substances. My research aimed to ascertain whether or not *J. mollissima* extract offered protection against the cardiotoxicity and nephrotoxicity caused by doxorubicin.

Excessive fluid collection in the pleural, pericardial, and peritoneal cavities indicated cardiomyopathy in my experimental models after intraperitoneal doxorubicin treatment (Herman et al., 1988). Due to aberrant cardiac functioning and the development of ascites, animals given doxorubicin had a 40% higher mortality rate than the control group by the end of the trial (Renu et al., 2018).

Before the termination of the experiment, a 40% mortality rate was observed in Doxo intoxicated group due to severe cardiac toxicity. As previously mentioned, ascites occurred due to tubular dysfunction and salt retention, which caused extracellular volume growth and fluid leaking towards the interstitium (Deschênes and Doucet, 2000). Multiple renal indicators pointed to nephrotoxicity, likely contributing to the high fatality rate. Due to the medications’ toxic effect on the intestinal mucosa, many people found they were eating less and losing weight. That was achieved by the drugs’ indirect effect on the digestive system, lowering release of endogenous hormones and reducing hunger (Zima et al., 2001). Previous reports indicated that using *Jm* led to increased food intake and improved intestinal mucosa, contributing to the rise in body weight compared to the intoxicated group (El-Far et al., 2016).

Cardiac cell apoptosis was due to reactive oxygen species (ROS) produced by DOX, which caused a damaging effect on lipids, DNA, and proteins and caused impaired cells (Abogmaza et al., 2020).

Creatine isoenzymes CK-MB, lactate dehydrogenase plus troponin I are the most reliable indicators of cardiac cell damage. Their level was highly elevated in the Doxo group, indicating severe cardiac cell damage. These markers were released due to oxidative damage of cardiac myocytes by Doxo, which caused the release of these cardiac biomarkers in the circulation (Abd-Allah et al., 2002). Increased formation of free radicals, especially superoxide anion, causes an inflammatory cascade. The heart is vulnerable to free radicals due to less free radical detoxifying substances like glutathione, catalase, and superoxidase dismutase (Olson and Mushlin, 1990).

The cardioprotective action of *Jm* was confirmed by the decrease in mortality rate and the reduction in CK-MB, troponin I, and LDH levels after administration of *J. mollissima* extract at doses of 400 & 600 mg/kg. It was suggested that *Jm* possesses anti-oxidant potential due to phenols, carotenoids, and anthocyanins (Pongprot et al., 2012). *Jm* possesses free radical scavenging activity causing a significant reduction in the lipid peroxide concentration, which gives protection from lipid peroxidation (Arafa et al., 2014). Also, doxorubicin has a high affinity to cardiolipin and phospholipids in cardiac tissues.

Increased plasma sodium levels were seen in doxorubicin-treated rats compared to controls, but these levels returned to baseline in *Jm*-treated groups. Increased sodium levels have also been reported in rats with cardiac damage (Oz and Ilhan, 2006).

As it had been previously investigated that cardiomyopathy caused hypokalemia, doxorubicin treatment decreased plasma potassium levels compared to the normal control group. In contrast, treatment with *Jm* increased potassium levels to almost normal values in the intoxicated group, as shown in earlier research (Stevens and Levey, 2005).

Doxorubicin was found to cause nephrotoxicity through oxidative stress, with the resulting free radicals leading to tubular atrophy and heightened glomerular capillary permeability. Damage to biological macromolecules and lipid peroxidation due to iron-dependent oxidative damage contributed to nephrotoxicity (Ma et al., 2013). Because doxo metabolites are partially excreted by the kidney, the progression of kidney disease is dose- and time-dependent. Renal injury can also be caused by the generation of hydroxyl radicals and superoxide anions due to the conversion of doxorubicin to semiquinone free radical by NADPH-Cytochrome P-450 (Yancy et al., 2013). Kidney and heart lesions and damage have been related to a number of drugs and environmental toxins (Abogmaza et al., 2020). Some drugs have been associated to shifts in cell and tissue lipid peroxides and metabolites. Pro-oxidants such as reactive oxygen (ROS) species can induce tissue damage (M. O. Iqbal and Yahya, 2021). Herbs with nephroprotective and cardioprotective properties abound, and these often include antioxidant-rich plants. Doxorubicin reduces acetyl-CoA concentrations, inhibits lipogenesis, inhibits glucose production, reduces urea production, and inhibits lipid peroxidation in rats. We found that *Jm* at both dosages (400 and 600 mg/kg) significantly (*p* > 0.005) mitigated the doxorubicin-induced oxidative stress and slowed the degradation of renal and heart function. Their ability to serve as radical scavengers and hydrogen donors is a result of the antioxidant strength provided by *Jm* phenols. Antioxidants found naturally in plants are secondary plant metabolites (Mosmann, 1983; Gülçin et al., 2004).

Due to impaired sodium reabsorption caused by tubular abnormalities, doxorubicin-treated groups excreted less sodium in their urine than the normal control group. Previous research revealed that activation of Na+/K+ ATPase in the cortical collecting duct was responsible for this sodium retention (Ahmed et al., 2019).

Sodium excretion was higher in *Jm*-treated groups, suggesting that the extract may have a positive effect on renal function thanks to *Jm*’s nephroprotective action, which consists of repairing proximal tubular damage with its antioxidative properties (Rodriguez de Sotillo and Hadley, 2002).

According to the most recent data (Mattiuzzi and Lippi, 2019) cancer is the second largest cause of death worldwide. It was responsible for an estimated 9.6 million deaths in 2018, up nearly 10% from 2015’s total of 8.8 million, according to (Demain and Vaishnav, 2011). This has a major impact on the global economy and patients’ quality of life.

Due to the global economic effect and the rising number of cancer fatalities, finding a cancer therapy has been the primary research focus. For over 40 years, oncology has benefited from using natural products (Qamar, Rehman and Chauhan, 2019). The researchers set out to answer the question, “Could a component of this plant be used as a treatment option for cancer patients?” and to build the groundwork for future research into the use of medicinal plants in cancer treatment.

In 2018, cancer was the second top killer worldwide. Several harmful effects have been linked to the rising use of chemotherapy and radiotherapy to increase cancer patient survival (Nayim et al., 2021). As a result, researchers have been looking for novel anticancer drugs that are more potent with fewer negative side effects. Recent research has shown that plant-based anticancer medications have minimal cytotoxic values in a dose-dependent MTT experiment, inhibiting tumor development without causing unacceptable side effects (Elmore, 2007). Thus, there has been a trend toward investigating the phytochemical content of medicinal plants in the quest for safe and effective cancer treatments.

The hepatocellular carcinoma (HepG2) cell line also showed a concentration-dependent response to the plant extracts. The highest concentration of plant extracts (400 g/mL) was shown to inhibit cells to a greater extent than any of the lower doses tested. As shown in the Figure 11, the percentage of cells inhibited rose with the concentration of the extracts. However, the effect on cell proliferation was minimal after 24 h of treatment with doses up to 90 g/mL of crude ethanolic extract in HepG2 cells. Cell viability declined by over 50% at doses higher than 100 μg/mL (Figure Figure11). Cell viability decreased proportionally after treatment with a different concentration of *Jm* extract. However, as Figure demonstrates, safe levels for healthy cells with a 90% or above viability are below 90 or 100 μg/mL. These results jived with a recently conducted study that evaluated the *in vivo* toxicological effect of the IC crude extract (Ho et al., 2009).

Therefore, determining which type of cell death is induced by the plant extracts, apoptosis or necrosis, is important. Apoptosis is characterized by the following events: cell detachment and shrinkage; extensive blebbing of the plasma membrane; and the formation of apoptotic bodies, which are composed of cytoplasm with tightly packed cellular organelles and may or may not contain nuclear fragments (Fesik, 2005). These entities are protected from lysis *in vivo* because macrophages and neighboring cells, including parenchymal and neoplastic cells, phagocytose them. In contrast, in an *in vitro* setting, the absence of phagocytes causes the apoptotic bodies to expand and lyse, leading to subsequent necrosis (Nicholson, 2000).

The ability of cells to avoid programmed cell death, often called apoptosis, is one of the six important modifications in cell physiology that control the growth of tumor cells (Van Engeland et al., 1998). This is because necrotic cells are less likely to undergo apoptosis than healthy cells, which can lead to uncontrolled tumor cell growth, failure to adapt to cellular stress, damaging mutations, and DNA damage (Moss et al., 1991).

The Annexin V-FITC Apoptosis detection kit is utilized in this study to identify the precise form of cell death caused by the bioactive plant extracts. The kit includes a mix of Annexin V, Propidium Iodide, and fluorescein isothiocyanate (FITC) conjugate to detect and differentiate apoptotic, necrotic, and dead cells. The negatively charged phospholipid phosphatidylserine (PS) is normally located in the cytosolic leaflet of the plasma membrane lipid bilayer. It was discovered by that annexin V binds to PS very strongly in the presence of physiological amounts of Calcium ions (Ca2+) (Moss et al., 1991; Kaufmann and Vaux, 2003), annexin V binds to PS during the transition from the inner to the outer leaflet of the plasma membrane during the early stages of apoptosis. When propidium iodide enters a cell and bonds to the DNA there, it shows that it is dead. The inability to induce programmed cell death (apoptosis) is a major obstacle to the effective use of anticancer drugs (Cummings et al., 2004).

It was determined that the phenols, flavonoids, and flavone in *J. mollissima* have anti-oxidant potential and can prevent doxorubicin-induced damage to the heart and kidneys.

## 5 Conclusion and recommendations

Based on the findings of this investigation, it appears that antioxidant enzymes could be normalized, cardiac enzymes could be improved, and pathways triggering cardiac apoptosis could be altered by co-administering *J. mollissima* extract with doxorubicin.

*Jatropha mollissima* hydroalcoholic extract also showed nephroprotective action by increasing the amount of free radical scavenging enzymes due to its anti-oxidant potential, captured free radicals, and reduced lipid peroxidation as it contains phenols and melatonin, which are free radical scavengers. Our results further demonstrated that *J. mollissima* ethanolic extracts are cytotoxic to the hepatocellular carcinoma (HepG2) cell line.

Therefore, it could be recommended that *J. mollissima* extract is effective against Doxo - induced cardiotoxicity and nephrotoxicity without attenuating the clinical efficacy of doxorubicin. To control Hepatocellular carcinoma, *Jm* is therefore considered a promising medicinal substance. The results indicated that the plant could inhibit tumor growth through various mechanisms (such as inducing apoptosis). The prevention of hepatocellular cancer has been greatly advanced due to this study. However, there is still a lot of research to be done to evaluate the relevant signaling pathway.

*Jatropha mollissima* extract could be used before treatment with doxorubicin or given during therapy to prevent cardiotoxicity and nephrotoxicity, evade the need for other medications, and thus improve the quality of the patient’s life.

## Data Availability

The original contributions presented in the study are included in the article/Supplementary Material, further inquiries can be directed to the corresponding authors.
